# Marriage, Cohabitation and Intimate Relationships in Later Life

**DOI:** 10.1093/geroni/igaf122.837

**Published:** 2025-12-31

**Authors:** Hui Liu, Sara Moorman

## Abstract

The five papers presented in this symposium utilize data from the National Social Life, Health, and Aging Project (NSHAP) to examine various types of intimate relationships in later life. Two papers focus on marital relationships. Li and Waite explore the likelihood of older adults naming their spouse or partner as a “core confidant” and how relationship quality influences this designation, finding that relationship quality significantly predicts spousal confidant status. Avent and Ivenuik examine how spousal retirement affects social networks and shared relationships, showing that retirement leads to more interconnected but smaller networks for men, while women’s networks initially grow before shrinking. The other three papers examine other intimate ties. Brown and Newmyer extend research on the marital biographies of older adults by incorporating cohabitation histories, offering a more comprehensive view of union experiences among U.S. older adults. Warner and Lyons investigate how couple-level sexual expression predicts subsequent loneliness for both spouses, showing that less-than-desired sexual activity is associated with greater loneliness, particularly among couples who view sex as an important part of life. Finally, Pope and Thomas move beyond human intimate ties to examine the impact of human-animal relationships on older adults’ well-being, finding that pet touch may benefit mental health of older adults, with effects varying by gender and marital satisfaction. Together, these five papers provide valuable insights into the diverse intimate ties that shape later life, including marriage, cohabitation, sexual expression, and human-animal relationships, and how these intimate ties influence the well-being of older adults. Human-Animal Interaction Interest Group Sponsored Symposium

